# The impact of the early environment on oxytocin receptor epigenetics and potential therapeutic implications

**DOI:** 10.1038/s41390-024-03563-z

**Published:** 2024-11-15

**Authors:** Madelyn G. Nance, Kelsey M. Sullivan, Meghan H. Puglia

**Affiliations:** 1 Department of Neurology, University of Virginia, Charlottesville, VA USA; 2 Department of Pediatrics, Division of Neonatology, University of Virginia, Charlottesville, VA USA

## Abstract

**Abstract:**

Oxytocin research is rapidly evolving and increasingly reveals that epigenetic modifications to the oxytocin receptor gene (*OXTR*) are functional, plastic, and reliable components of oxytocinergic system function. This review outlines how *OXTR* epigenetics are shaped by the early life environment, impact social-developmental outcomes, and have strong potential to serve as therapeutic targets. We first establish the malleability of *OXTR* epigenetics in infancy in both animal models and humans through research demonstrating the impact of the early life environment on *OXTR* DNA methylation (*OXTR*m*)* and subsequent social behavior. Next, we detail how *OXTR*m serves as a predictive mechanism for neurodevelopmental outcomes in animal models of social behavior such as the prairie vole, and summarize the role of *OXTR*m in psychiatric disorders, emotional processing, and attachment behavior in humans. We discuss the potential of further *OXTR*m research to improve oxytocin therapeutics by highlighting how a deeper knowledge of *OXTR*m could improve the therapeutic potential of exogenous oxytocin, how *OXTR*m may impact additional cellular mechanisms with therapeutic potential including control of the perinatal GABA switch, and how early life therapies may target the tuning of endogenous *OXTR*m. Finally, we review limitations of previous oxytocin research and make recommendations for future research.

**Impact:**

- Previous research into oxytocin therapeutics has been hampered by methodological difficulties that may be improved by assay of the oxytocin receptor gene (*OXTR)* and its methylation (*OXTR*m)
- Key sites of *OXTR*m modification link early life exposures to developmental and behavioral outcomes
- *OXTR*m appears to have a critical period of development in early life
- Epigenetic modification of the oxytocin receptor gene could serve as a powerful target for therapeutic interventions

## Introduction

**Fig. 1:**
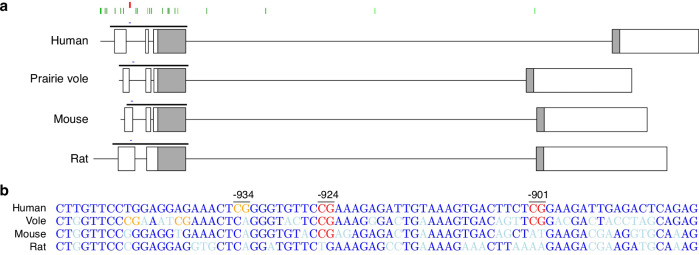
Structure and conservation of the oxytocin receptor gene. **a** A schematic of the human, prairie vole, mouse, and rat oxytocin receptor gene (OXTR). Lines represent introns and boxes represent exons (grey = coding regions; white = untranslated regions). The black bar above each gene schematic depicts the location of a CpG island, and the blue bar depicts the location of the genetic sequences specified in (**b**). Above the human gene, green lines indicate the location of CpG sites queried by the Illumnia array (dark green=sites on Illumina 450k array; light green = additional sites added to the Illumina 850k array), and red lines indicate the location of CpG sites -934, -924, and -901. **b** Alignment of the human, prairie vole, mouse, and rat gene sequence for a region of the OXTR promoter containing CpG sites -934, -924, and -901 sites. Residues in dark blue are conserved across human and animal. Residues in light blue are not conserved. CpG sites in red are conserved across human and animal. CpG sites in orange are putatively conserved across human and animal. Human: hg38_dna range = chr3:8769072-8769140; Prairie vole: hub_2175119_GCF_000317375.1_dna range = NW_004949099.1:26357588-26357658; Mouse: mm10_dna range = chr6:112490614- 112490683 ; Rat: rn6_dna range = chr4:144416937144417001.

**Fig. 2:**
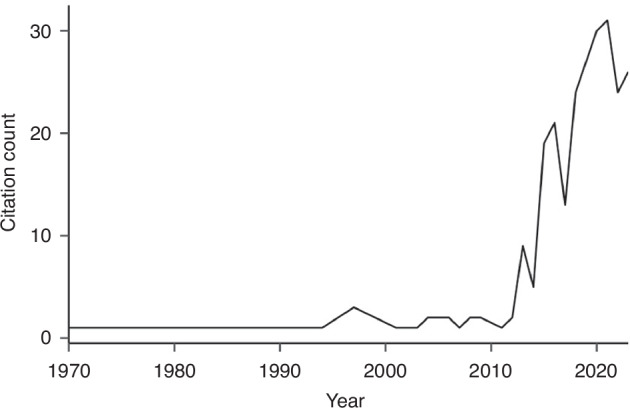
Citations related to oxytocin receptor methylation over time. Using a bibliometric analysis tool, we detected a 44% average increase in research pertaining to oxytocin receptor methylation in the past 10 years.

Oxytocin is a highly conserved neuropeptide with effects ranging from birth mechanisms to social behavior. Since its discovery, this pleiotropic molecule has been the topic of extensive research.^1^ Early oxytocin studies investigated its role in the timing of birth and the initiation of maternal behavior.^2,3^ Subsequent work has highlighted oxytocin’s role in both social behavior and psychopathology.^4–11^ However, the results of prior research into the oxytocin hormone itself and its clinical applicability have been highly variable, in part due to oxytocin’s short half-life and high binding affinity to other proteins which make it difficult to reliably assay the protein.^12^ The actions of oxytocin are dependent upon the availability of its receptor, OXTR, to bind oxytocin and initiate intracellular signaling processes. Consideration of epigenetic modifications to the oxytocin receptor gene (*OXTR*,^1^ Fig. 1) promises to add to our understanding of the oxytocinergic system’s function and plasticity with greater reliability.^12^ Recently, oxytocin research has increasingly expanded (Fig. 2^13^) to include such epigenetic modifications, specifically DNA methylation of the oxytocin receptor gene (*OXTR*m).

DNA methylation is a tissue-specific epigenetic modification in which a methyl group is added to the fifth carbon of a cytosine nucleotide when adjacent to a guanine nucleotide (CpG sites).^14^ DNA methylation most commonly occurs in CpG islands – regions that have a high quantity of cytosine-guanine dinucleotides and frequently occur in the promoter regions of a gene.^15^ Methylation of promoter regions can lead to an inability to initiate transcription,^15^ resulting in transcriptional dampening and decreased gene expression.^16,17^

**Fig. 3:**
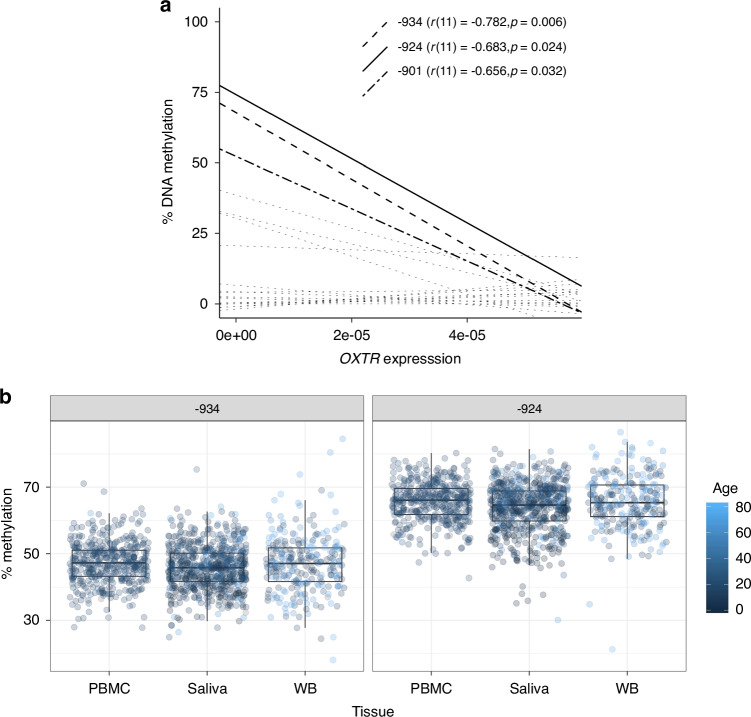
CpG Sites -934 and -924 are functional and variable. **a** The association between OXTR methylation and expression in human temporal cortex for every CpG site within the OXTR MT2 promoter region. Only functional sites -934, -924, and -901 show significant associations between methylation and expression. Data adapted from ref. ^19^, with permission from Springer. **b** OXTR methylation at sites -934 and -924 is highly variable across individuals. Each point depicts a unique sample (*n* = 1681) assayed in our lab from peripheral blood mononuclear cells (PBMC), saliva, and whole blood (WB) from individuals ranging in age from 0 days to 81 years. Younger ages are depicted in darker colors.

In one of the most critical early investigations into *OXTR*m, Gregory, Connelly and colleagues assayed all CpG sites within a region previously identified to impact *OXTR* transcription, called MT2,^18^ to identify the specific sites that showed a significant association between *OXTR*m and *OXTR* expression in the human temporal cortex. They identified only 3 CpG sites in intron 1, -934, -924, and -901, which show a significant association between methylation and gene transcription in the brain^16^ (Fig. 3a^19^) – the causal tissue for behavior. Increased levels of methylation at these functional sites are linked to decreased transcription of the gene^16^ and thus presumably decreased ability to bind and use oxytocin. Additionally, methylation levels at these sites are highly variable across individuals (Fig. 3b), highlighting their promise to serve as markers of individual differences in (endo)phenotypes. Finally, *OXTR*m at these sites is correlated across cell^20^ and tissue^19,21–24^ types, and critically, methylation levels in both cortical and peripheral tissues respond to early-life environmental manipulations in a similar manner.^16,19,24^ Thus, *OXTR*m can be assayed noninvasively from easily accessible tissues such as saliva to be informative of methylation levels in the brain that are plastic and are promising targets of therapeutic interventions.

Recent reviews have summarized how epigenetic alterations to *OXTR* contribute to differences in social behavior and pathology in humans^10,11,25^ and animals.^26^ Here, we are the first to consider a cross-species developmental understanding of the impact of *OXTR*m from the social brain to the neuronal membrane, as well as its potential as a therapeutic target. This review will outline recent advances in the field of oxytocin epigenetics to illustrate its candidacy for therapeutic research. First, we review recent literature demonstrating that oxytocin receptor epigenetics are malleable in early life. Next, we present evidence that oxytocin receptor epigenetics are related to subsequent neurodevelopmental outcomes. Finally, we review evidence supporting oxytocin receptor epigenetics as a powerful therapeutic target. We conclude with a discussion on the limitations of previous oxytocin research and recommendations for future research. A summary of reviewed studies is available in Table 1.

**Table 1:** Summary of studies cited in this review, including brief descriptions of the target population, the tissue studied, the ages of the research subjects, the molecular site studied, the main finding of each study, and the effect size.

Publication	Population	Tissue	Age	Site	Main Finding	Effect Size
Gregory et al.^16^	Human	Temporal Cortex	5–30 years	-934, -924, -901	Only 3 CpG sites are functional, informative of oxytocinergic system function, and associated with decreased gene transcription. *OXTR* is hypermethylated most prominently at sites -924 and -934 in the peripheral blood of unrelated probands with autism spectrum disorder (ASD).	Not Reported
		Peripheral Blood Mononuclear Cells (PBMC)				
Beery et al.^38^	Rat	Hippocampus, Striatum, Hypothalamus	15–16 weeks	25 CpGs within promoter	Decreased maternal care is correlated with increased *Oxtr*m.	Not Reported
		PBMC				
Perkeybile et al.^24^	Prairie Vole	Nucleus Accumbens	PND0, PND24	-934_1, -934_2, -924, -901	Decreased early parental care is associated with increased Oxtr methylation in both blood and brain tissue.	Not Reported
		Whole Blood	PND24			
Danoff et al.^19^	Prairie Vole	Nucleus Accumbens	PND0, PND24	All sites in MT2 and exon 3	Decreased early parental care is associated with increased methylation of Oxtr in MT2 and exon 3.	The effect size of the handling condition on the standardized network community score (representation of DNA methylation values) is largest in community 3 (sites 924 and 934). *d* = 1.42
		Whole Blood	PND24			
Unternaehrer et al.^41^	Human	Whole Blood	Adults	OXTR_TS1_, OXTR_TS2_	Low maternal care conditions are associated with increased methylation of OXTR in exon 3.	Not Reported
King et al.^44^	Human	Saliva	Adults (M = 35.52 years)	22 sites on a CpG island on exon 3	Infants exposed to maternal depression had increased *OXTR*m with the highest methylation values in infants exposed to persistent maternal depression.	Not Reported
			Toddlers (M = 2.9 years)			
Fujisawa et al.^45^	Human	Saliva	Children (M = 12.9 years)	20/27 sites in MT2	Children who experience childhood maltreatment have increased *OXTR*m in saliva and increased rates of insecure attachment.	Not Reported
Krol et al.^21^	Human	Saliva	5 and 18 months	-924	In infancy, *OXTR*m and maternal care at 5 months of age is predictive of changes in *OXTR*m at 18 months of age.	Not Reported
Krol et al.^22^	Human	Saliva	5 months	-924	High levels of *OXTR*m at 5 months of age is associated with increased inferior frontal cortex activity to angry or fearful facial expressions in comparison to happy faces at 7 months of age.	Not Reported
Puglia et al.^23^	Human	Saliva	5 months	-934	Infants with lower *OXTR*m have increased neural variability and improved behavioral scores by parent report.	*f*^2^ = 0.07 and *f*^2^ = 0.10 respectively.
Puglia et al.^20^	Human	PBMC	18–30 years	-934	Increases in *OXTR*m at functional sites are correlated with increased neural response in the brain regions associated with regulating the salience of stimuli and attention.	Not Reported
MacKinnon et al.^47^	Human	Saliva	2–3 years	22 CpG sites on exon 3	Increased levels of *OXTR*m were associated with decreased performance on theory of mind tasks.	Not Reported
Jack et al.^52^	Human	PBMC	18–30 years	-934	Observed a positive interaction between *OXTR*m and neural activity when viewing shapes interacting implying animacy or shapes displaying random movement.	Not Reported
Puglia et al.^20^	Human	PBMC	18–30 years	-934	Increased levels of *OXTR*m are associated with increased neural activity in areas required for human face processing and emotional regulation.	Not Reported, sample size too small
Skyberg et al.^51^	Human	Saliva	5–11 years	-924	Observed a positive association between increased *OXTR*m and increased activity in brain regions associated with social skills.	Not reported
Skyberg et al.^48^	Human	Saliva	5–11 years	-934	Observed a positive association between increased OXTRm and more precocious maturation of brain circuitry related to emotion and sociality.	Not Reported
Rijlaarsdam et al.^53^	Human	Cord blood	Birth	OXTR CpG island; hg19; chr3:8808962–8811280)	Associations between OXTRm and ASD are dependent on a highly investigated OXTR genotype, rs53576	Not Reported
Dadds et al.^99^	Human	Blood	4–16 years	11 CpG dinucleotides within a CpG island within the promoter	Observed increased *OXTR*m and decreased basal oxytocin in children with increased callous-unemotional traits and decreased empathy.	Not Reported
Cecil et al.^55^	Human	Cord blood, Blood	Birth, 7 and 9 years at blood collection	11 CpG dinucleotides within a CpG island within the promoter, same as in Dadds et al.	In participants with low internalizing symptoms, there was an association between increased *OXTR*m at birth and prenatal risk.	Not Reported
Milaniak et al.^65^	Human	Cord blood	4–13 years	12 probes within the CpG island	Children with increased *OXTR*m levels display increased resilience to the development of conduct disorders.	Not reported
Aghajani et al.^56^	Human	Saliva	15–19 years	Exon 3	Relative to healthy controls, higher levels of *OXTR*m and callous unemotional traits interact to predict increased activity and increased disconnection between brain regions when observing distressed facial expressions.	Not Reported
Rubin et al.^57^	Human	Whole blood	Not reported	-934	Increased levels of *OXTR*m are associated with poorer emotion recognition in both women with psychotic disorders and healthy control women.	Not Reported
Gordon et al.^87^	Human	N/A	8–16.5 years	N/A	Patients with ASD given a single dose of exogenous intranasal oxytocin had increased neural activity in key brain regions during social judgment making.	Not Reported
Domes et al.^88^	Human	N/A	Adults	N/A	Increased amygdala activity was observed in response to social stimuli when individuals with ASD were administered a single dose of intranasal oxytocin.	Not Reported
Watanbe et al.^90^	Human	N/A	18–55 years	N/A	After 6 weeks of oxytocin administration in ASD participants, there were significant reductions in their autism diagnostic observation schedule scores (ADOS).	Effect on ADOS reciprocity score: *d* = 0.78 Effect on ADOS communication score: d = 0.03 Effect on ADOS repetitive behavior score: *d* = 0.24
Tachibana et al.^92^	Human	N/A	10–14 years	N/A	When intranasal oxytocin was administered to a group with ASD with dosage increases every 2 months) the majority had improved ADOS scores.	Not Reported
Bales et al.^93^	Prairie Vole	N/A	21–60 days	N/A	When administering three doses of oxytocin from postnatal day 21 to 42 there was an initial increase in male social behavior however, eventually partner preference waned.	Not Reported
Goldman et al.^94^	Human	N/A	Not reported	N/A	In patients with schizophrenia, lower doses of oxytocin increase emotion recognition, while higher doses have the opposite effect.	Not Reported
Hall et al.^95^	Human	N/A	13–28 years	N/A	Males with Fragile X syndrome receiving lower doses of oxytocin have increased frequency of eye gaze however, high doses of oxytocin return eye gaze frequency to baseline.	Not Reported
Dadds et al.^54^	Human	N/A	7–16 years	N/A	No difference in social behavior between those who received intranasal oxytocin as compared to placebo.	Not Reported
Guastella et al.^100^	Human	N/A	12–18 years	N/A	No difference in social behavior between those who received intranasal oxytocin as compared to placebo.	Effect of oxytocin treatment on SRS score posttreatment: *d* = 0.06 Effect of oxytocin treatment on SRS score at 3-month follow-up: *d* = 0.03
Mairesse et al.^105^	Rat	Brain - microglial cells	Conception - postnatal day 2 or 4	N/A	Microglial activation of Oxtr regulates inflammation in the neonatal brain.	Not Reported
	Zebrafish		8 days postfertilization			
Ceanga et al.^104^	Rat	Brain - hippocampus	Postnatal day 0	N/A	The viability of neural tissue is improved when incubated with oxytocin under hypoxic conditions, an effect not observed when Oxtr was blocked with an antagonist.	Not Reported
Tyzio et al.^110^	Mouse	Brain - hippocampus	Embryonic days 20–21, postnatal days 15–30	N/A	The GABA switch is completely absent in two different mouse models of ASD. When the switch is restored the behavioral phenotype is restored.	Not reported
Bertoni et al.^113^	Mouse	Brain - Hippocampal neurons	Embryonic day 18	N/A	A delay in the GABA switch disrupts social memory and this effect is reversed with neonatal application of oxytocin.	Not Reported
Robakis et al.^126^	Human	Buccal cells	>18 years	151 CpG sites	Out of over 1,000 regions assayed for association with early life adverse experiences none were associated with psychological outcomes.	Not Reported
Lesemann et al.^127^	Human	Saliva	Adolescents - adults	-934	*OXTR*m is associated with decreased neural activity in response to facial expressions. However, this effect does not survive statistical correction.	Not Reported
Siu et al.^64^	Human	Whole blood for ASD cohort	2–18 years	-989, -982, -959, -934, -924, -860, -835, -826	Males with ASD had *OXTR* hypomethylation at site -982 compared to controls. A mixed-sex comparison of individuals with ADHD had decreased *OXTR*m at sites -934 and -924 compared to controls. A mixed-sex comparison of individuals with OCD had decreased *OXTR*m at sites -835 and -826 compared to controls.	1.73% decrease of *OXTR*m at site -982 in males with ASD 1.65% and 4.95% decrease in *OXTR*m at sites -934 and -924 respectively in individuals with ADHD 1.37% and 1.62% decrease in *OXTR*m at sites -835 and -826 respectively in individuals with OCD
		Saliva for ADHD and OCD cohorts				
Kenkel et al.^40^	Prairie vole	Paraventricular nucleus, supraoptic nucleus, hypothalamus, amygdala, parietal cortex	Expected day of delivery for fetal voles - adulthood	-934_1, -934_2, -924, -901	Maternally-administered intrapartum OXT leads to a dose-dependent increase in *Oxtr*m in the fetus and adult offspring. These changes are also correlated with more gregarious social behavior in OXT-exposed offspring.	Not reported

## Oxytocin receptor epigenetics are malleable in early development

### Animal models demonstrate early *Oxtr*m malleability

A substantial body of literature has established a causal relationship between increased caregiving behaviors in rodents and increased *Oxtr* expression, Oxtr protein density, and oxytocin binding in key brain areas across the lifespan.^27–29^ These alterations in caregiving behaviors^30–33^ and associated brain changes^27–29,32^ are correlated with resilience^30,32,34^ and social-emotional behavior such as aggression and parenting behaviors^31,33^ later in life.

The prairie vole (*Microtus ochrogaster*) is an optimal phenotypic and genetic animal model for understanding social behavior^8,35^ and *Oxtr*m. These animals are highly social, forming life-long monogamous pair bonds where both parents actively engage in parenting responsibilities.^8^ Additionally, the promoter region of *Oxtr*^19^ is highly conserved across prairie voles and humans, including sites -934, -924, and -901, the last of which is functionally related to -934_1 and -934_2 in prairie voles^24^ (Fig. 1). All CpG sites that show a significant association between DNA methylation and gene expression in human cortex are conserved in prairie vole *Oxtr*, whereas only one residue (-924) is conserved in mouse *Oxtr*^36^ and none are conserved in rat *Oxtr*^37^ (Fig. 1b).

An early study by Beery et al. demonstrated that decreased maternal care correlated with increased *Oxtr*m in rat peripheral blood mononuclear cells across numerous (non-conserved) CpG sites.^38^ This association between maternal care and *Oxtr*m was replicated and furthered by Perkeybile et al., who examined the relationship between caregiving behavior and *Oxtr*m in prairie voles.^24^ Perkeybile’s study proposed an epigenetic mechanism whereby decreased early parental care leads to increased *Oxtr*m across all conserved and assayed CpG sites (934_1, -934_2, -924, -901) in whole blood and decreased Oxtr expression in the nucleus accumbens.^24^ In this study, prairie vole breeding pairs were either handled once on the day of parturition or not at all, and parental behaviors were quantified in the two groups. This early handling manipulation increases parental care immediately after intervention.^29,39^ Perkeybile et al. demonstrated that pups raised by the handled–or increased parental care–group had decreased blood and brain *Oxtr*m compared to pups raised by the unhandled–or decreased parental care group.^24^ Additionally, the pups of handled and unhandled dams were compared to term pups euthanized prior to any parental care. The pups of unhandled dams had increased *Oxtr*m compared to those euthanized prior to parental care, suggesting that decreased parental care causes de novo methylation of *Oxtr*.^24^ This group recently expanded this work to include another conserved and commonly investigated region of *Oxtr* on exon 3. While the finding that increased *Oxtrm* is correlated with low parental care was replicated in both regions, methylation was functional (i.e., showed associations with gene expression) only in the promoter sites (-934, -924, and -901 sites).^19^ These results are highly relevant to the study of human epigenetics given that these sites are conserved in human *OXTR*, are hypermethylated in pathologic states,^16^ and are known sites of epigenetic modification in response to parental care,^21^ as will be described in further detail later in this review.

Animal research has also demonstrated that differential exposure to oxytocin early in life results in differential *Oxtr*m.^40^ This is a particularly important avenue of investigation as oxytocin (e.g., Pitocin^®^) is commonly used in labor and delivery and conventionally believed to have no impact on the fetus or neonate. However, a recent study found that oxytocin administered to the pregnant dam on the expected day of delivery resulted in increased fetal brain *Oxtr*m. Oxytocin-exposed pups also demonstrated increased social behavior as adults.^40^ This study further serves to underline the early malleability of *Oxtr*m in response to oxytocin exposure and its impacts on later behavioral outcomes.

### *OXTR*m is malleable in early human development

An early study examining the impact of maternal care on *OXTR*m in humans measured two target sequences of *OXTR* in peripheral whole blood to understand the epigenetic impact of maternal care.^41^ In this study, adults were selected based on either a low or high maternal care sub-score of the Parental Bonding Instrument, a retrospective self-report questionnaire on perceived care and protection provided during the first 16 years of life.^42^ Low maternal care scores were associated with increased methylation of an *OXTR* site on exon 3.^41^ Associations between maternal care and offspring *OXTR*m were also demonstrated by King et al., who analyzed maternal and infant salivary *OXTR*m from mother-infant dyads and surveyed the mothers for postpartum depression with the Edinburgh Postnatal Depression Scale (EPDS).^43^ They found that infants exposed to maternal depression had increased *OXTR*m, with the highest methylation levels in infants exposed to persistent maternal depression.^44^ Likewise, Fujisawa et al. described that children who experienced childhood maltreatment showed both increased *OXTR*m in saliva and higher amounts of insecure attachment styles.^45^

A particularly important longitudinal study demonstrating early plasticity of *OXTR*m in infancy first examined maternal and infant *OXTR*m and maternal engagement at 5 months of age, and then assessed *OXTR*m in the dyad at 18 months of age, along with a child temperament assessment.^21^ The findings were consistent with previous studies that showed maternal care associations with *OXTR*m. The results demonstrated an average decrease in *OXTR*m from 5 to 18 months in infants, with a more pronounced decrease in infants exposed to increased maternal engagement.^21^ Specifically, *OXTR* site -924 was shown to change significantly in response to maternal care.^21^ Importantly, maternal *OXTR*m was relatively stable over the measured time period, suggesting a likely critical period for the development and calibration of the endogenous oxytocin system in infancy.^21^

## *OXTR*m influences subsequent neurodevelopmental outcomes

**Fig. 4:**
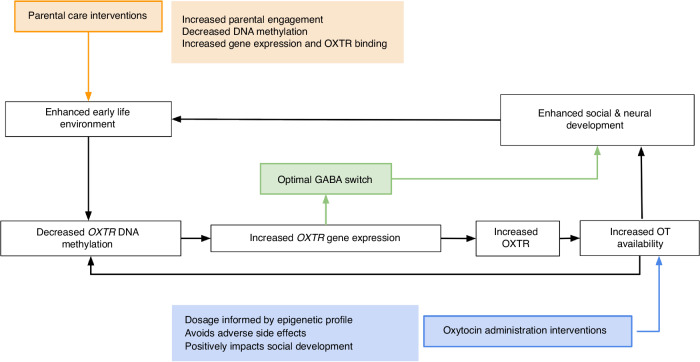
Proposed association between the early life environment, OXTR DNA methylation, and social and neural development. Lower levels of OXTR DNA methylation are associated with increased OXTR transcription and presumably increased ability to bind and use oxytocin. We posit that an enhanced early life environment optimizes social and neural development through precisely timed initiation of the “GABA switch” and increased sensitivity to oxytocin through decreased OXTR methylation. Enhanced social development and its associated neural specialization supports increased positive experience with the social environment, which further enhances molecular signaling. Parental care interventions and/or oxytocin administration interventions informed by epigenetic profiles have potential to improve psychopathology associated with altered OXTR methylation.

We posit that successful social development may emerge through a cyclical trajectory (Fig. 4) whereby biological predisposition (e.g., low *OXTR*m) establishes early attentional biases for social information, thus increasing experience with social information, which facilitates neural specialization and enhances molecular signaling, further driving perceptual experience with social information and fine-tuning the neural and molecular systems that support social functions throughout the lifespan. For example, Puglia et al. found a significant association between neural signal variability during social perception, *OXTR*m, and emergent social behavior in infancy.^23^ Decreased *OXTR*m at site -934 was associated with increased neural variability, specifically during social perception, and improved social, but not nonsocial, behavior. The authors hypothesized that this socially-evoked brain signal variability may serve to increase the salience of social information for these babies with low *OXTR*m, setting off a cascade of events for optimal social neurodevelopment. The specificity of these results highlights the importance of considering methodological factors such as task and neural region when investigating a role for *OXTR*m in socio-emotional development.

The importance of methodological considerations is further highlighted in a study investigating associations between *OXTR*m and neural response during emotional processing.^22^ This study examined salivary *OXTR*m in infants at 5 months and inferior frontal cortex (IFC) activity while looking at facial expressions at 7 months. The group found differential infant responsiveness to emotional facial expressions based on the infant’s *OXTR*m.^22^ Decreased *OXTR*m at site -924 was correlated with decreased IFC responsiveness to angry and fearful facial expressions – a finding also replicated in an adult fMRI sample^22^ – but increased responsiveness to happy facial expressions.

The general trend of these results suggests that decreased *OXTR*m has a positive effect on positive social behavior, specifically. In another study,^20^ site -924 *OXTR*m was associated with a sub-score on the Early Childhood Behavior Questionnaire^46^ such that 18-month-olds with decreased *OXTR*m displayed decreased temperamental discomfort. MacKinnon et al. also observed negative associations between *OXTR*m and performance on theory of mind tasks in children^47^ such that those with decreased *OXTR*m performed better on these tasks. Skyberg et al., however, found that higher levels of site -934 *OXTR*m were associated with enhanced socio-emotional functioning in children aged 5–11.^48^ These results were driven by more mature development in resting-state brain circuitry related to emotion regulation. The authors reconcile these seemingly discrepant results through the lens of the stress acceleration hypothesis^49^ which posits that precocious development is an adaptive mechanism to benefit those raised in a stressful environment. These results again highlight the need for longitudinal investigations that consider the nuanced interactions between molecular and environmental factors shaping developing neural circuitry and subsequent neurodevelopmental outcomes.

Another body of literature suggests that decreased sensitivity to endogenous oxytocin may necessitate compensatory neural mechanisms for successful social functioning throughout the life span. Indeed, an early study examining the relationship between *OXTR*m and selective social attention in adulthood showed increased *OXTR*m at site -934 was correlated with increased neural response in brain regions associated with regulating salience of stimuli and attention.^20^ Similarly, Puglia et al. found a significant association between increased levels of site -934 *OXTR*m and increased neural activity during the perception of anger and fear in areas required for the processing of human faces and emotional regulation in adults.^50^ Finally, a significant association has been demonstrated between increased *OXTR*m and increased neural activity during the perception of animate motion at sites -924 in children^51^ and -934 in adults.^52^ These findings further support the hypothesis that *OXTR*m is involved in promoting attention to social information to influence normative social-emotional processing throughout the lifespan.

## *OXTR*m is implicated in psychiatric disorders

*OXTR*’s role in the early establishment of social behavior^21–23^ and normative social processing^47,50,52^ may help explain its role in predicting later psychopathologies.^16,53–57^ It has repeatedly been shown that disordered or maladaptive changes in *OXTR*m are associated with the pathologic states of social, developmental, and psychiatric disorders. For example, autism spectrum disorder (ASD) is a common neurodevelopmental disorder characterized by social and language delays, as well as restricted and repetitive thought processes and behaviors.^44^ ASD first became a subject of interest in epigenetics due to its high heritability^58,59^ despite the fact that a minor portion of that heritability is due to genetic sequence variation.^16,60,61^ A landmark study examining the relationship between *OXTR*m and social processing revealed ASD-associated hypermethylation of *OXTR* sites -934, -924, -901, and -860 in temporal cortex, and sites -959, -934 and -860 in peripheral blood.^16^ This ASD-associated hypermethylation was later replicated in an independent study investigating *OXTR*m in saliva.^62^

However, two other studies failed to directly replicate this result. Elagoz Yuksel et al. found no association with *OXTR*m in the region assayed by Gregory et al., and ASD-associated hypomethylation in other regions of *OXTR* in peripheral blood in childhood.^63^ Siu et al. also found general hypomethylation of peripheral blood at one site (-982) in male children with ASD, but also that a substantial portion of the ASD cohort had extremely hypermethylated values.^64^ Discrepancies such as these highlight both the importance of methodological considerations such as locus, tissue, and age for epigenetic research,^11^ as well as consideration of the multifaceted underlying etiologies of complex disorders like ASD that require a longitudinal consideration of multiple genetic, epigenetic, and environmental factors. For example, in 2017, Rijlaarsdam et al. found that associations between *OXTR*m and ASD were dependent on a highly investigated *OXTR* genotype, rs53576.^53^ Collectively, these findings support the hypothesis that abnormalities in *OXTR*m are associated with ASD risk.

In adolescents, increased *OXTR*m has also been associated with callous-unemotional (CU) traits, which are associated with the development of psychopathy in adulthood.^54,55^ Dadds et al. tested peripheral blood *OXTR*m in children and adolescents diagnosed with oppositional defiant disorder or conduct disorder and found increased *OXTR*m and decreased basal oxytocin in children with increased CU traits and decreased empathy.^54^ Similarly, in a 13-year longitudinal study, Cecil et al. examined cord blood *OXTR*m at birth and peripheral blood in childhood in patients with CU traits. Participants with low internalizing symptoms demonstrated an association between increased *OXTR*m at birth and prenatal risks such as maternal psychopathology, criminal behavior, and substance use. The increased *OXTR*m levels persisted through age nine.^55^ Another analysis of the same cohort revealed increased resilience to development of conduct disorders in middle childhood in children with increased *OXTR*m.^65^ Aghajani et al. furthered these findings in a study that examined the interaction between *OXTR*m and CU traits on brain activity in juvenile offenders with conduct disorder versus matched healthy controls when viewing distressing facial expressions.^56^ The two groups did not differ in *OXTR*m levels, but the interaction of *OXTR*m and CU traits were associated with hyperactivity in key socio-affective brain regions.^56^ These findings provide evidence supporting a role of *OXTR*m in processing social-emotional information.

In addition to ASD and CU traits, *OXTR*m likely plays a role in many other pathologies, including psychotic and mood disorders. *OXTR* has been identified as a potential mediator in schizophrenia and other psychotic disorders. Rubin et al. examined the association between whole blood *OXTR*m at site -934, facial emotion recognition, and volumes of brain regions in participants with psychotic disorders compared to healthy controls.^57^ They found that increased *OXTR*m was associated with poorer emotion recognition in women with psychotic disorders and in healthy control women.^57^

Finally, increased *OXTR*m and its interactions with genotype and other risk factors has been associated with an array of mood and mood-related disorders, including depression and anxiety,^66–69^ social anxiety,^70^ postpartum depression,^44,71,72^ obsessive-compulsive disorder,^64,73^ anorexia nervosa,^74,75^ attention deficit and hyperactivity disorder,^64^ and post-traumatic stress disorder.^76^ The altered oxytocinergic system’s role in various psychopathologies, its accessibility,^20–23^ and its plasticity in response to the environment^12,21^ make it an attractive target for therapeutics.

## *OXTR*m is a potential therapeutic target

For 70 years, synthetic oxytocin (e.g., Pitocin^®^) has been used therapeutically, most commonly to augment labor and prevent postpartum hemorrhage.^77,78^ By the early 1990s, oxytocin was increasingly being used to augment social responses and receptiveness.^79^ Since then, many studies have examined the impact of exogenous oxytocin on social processing and psychiatric disorders.^62,79–85^ However, the actions of oxytocin are dependent on its receptor, making an understanding of the mechanisms driving receptor availability, such as *OXTR* methylation, critical to the field of therapeutics.^86^

In this section, we outline *OXTR*m’s therapeutic potential in the social brain, including a discussion of previous oxytocin therapeutic research in social processing, potential mechanisms explaining the benefit of oxytocin, and how emerging research targeting *OXTR*m may open new therapeutic avenues.

### Oxytocin therapeutics in the social brain

#### Intranasal oxytocin impacts social processing

Given the likely role of disordered oxytocinergic system regulation in social-developmental disorders, application of intranasal oxytocin has been extensively studied and shows promise for treatment, albeit with mixed results. Gordon et al. found that giving patients with ASD a single dose of exogenous oxytocin increased neural activity in key brain regions when making social judgments.^87^ In a similar study, Domes et al. found increased amygdala activity in response to social stimuli when individuals with ASD were administered a single dose of intranasal oxytocin.^88^ Likewise, chronic oxytocin administration shows promise in improving social brain function. In a study of adults with ASD, participants significantly reduced their scores on the Autism Diagnostic Observation Schedule (ADOS)^89^ after 6 weeks of oxytocin administration,^90^ though in this case the changes were not significantly increased as compared to single-dose administration.^91^ In an open-label study, Tachibana et al. administered intranasal oxytocin to a group of eight participants with ASD, increasing the dosage every 2 months. Six out of eight participants had improved ADOS scores at the end of the study period.^92^

However, limitations of exogenous oxytocin application include mixed reported effects, narrow study populations, and variable manner of delivery. For example, Bales et al. found that when administering three doses of oxytocin from postnatal day 21–42, there was an initial increase in social behavior in male prairie voles; however, following chronic administration, partner preference waned.^93^ The authors theorized that repeated stimulation of Oxtr results in its sequestration and decreased ability to bind oxytocin. This hypothesis was supported by a study that found that within 10 minutes of OXTR agonist stimulation, over 60% of receptors on human embryonic kidney cells were internalized and did not return to the cell surface.^1^

The literature reveals similarly paradoxical effects in humans. In a study of patients with schizophrenia, lower doses of oxytocin increased emotion recognition, while higher doses reversed this effect.^94^ Similarly, Hall et al. found that males with Fragile X syndrome, a genetic disorder highly comorbid with ASD, responded to oxytocin in a dose-dependent manner.^95^ Patients receiving lower doses of oxytocin exhibited increased frequency of eye gaze; however, when the dose of oxytocin was doubled, eye gaze frequency returned to baseline.^95^ These divergent effects could be explained by receptor sequestration. It is important when discussing intranasal oxytocin research to understand that there are many reviews which summarize the mixed effects and concerning statistical trends. Quintana et al. interrogate the biological bases of intranasal oxytocin application including our limited understanding of nose-to-brain drug delivery and penetrance.^96^ Mierop et al. point out the heterogeneous effects of these studies, their absent or failed replications, and many broad statistical issues.^97^ Finally, we point the reader to Walum et al. who outline and explain the issues of insufficient power that are seen in many meta-analyses of intranasal oxytocin research.^98^

#### Incorporating *OXTR* epigenetics into therapeutic research may enhance outcomes

Paradoxical therapeutic effects of oxytocin administration are further complicated by the epigenetic mechanisms of *OXTR* regulation. Those with low *OXTR*m could have an increased tolerance for higher doses of oxytocin and may not experience adverse decreases in sociality. Those with high *OXTR*m may not be primed to bind and use oxytocin effectively or may co-activate related systems (e.g., vasopressin) and therefore experience adverse effects. The dose-dependent effects of oxytocin administration potentially explained through sequestration could in itself be regulated by individual differences in baseline expression of *OXTR*. Examples of mixed results in oxytocin therapeutic research underline the possibility that study designs informed by *OXTR*m could enhance future research. For example, two randomized control trials found no difference in social behaviors in participants who received intranasal oxytocin as compared to placebo.^99,100^ Additionally, some trials have been associated with adverse effects including aggression and gastrointestinal discomfort.^101^ Without an understanding of participants’ baseline *OXTR*m, which is often highly variable (Fig. 3b), these effects are impossible to distinguish.

The studies outlined in this section point to a need for studying *OXTR* epigenetics in tandem with exogenous oxytocin administration. A deeper understanding of the role of epigenetics in the oxytocinergic system could help limit adverse side effects, mixed behavioral responses, and inform a precision-medicine approach to oxytocin administration. Using precision medicine to inform oxytocin administration may have a particular therapeutic benefit for younger populations in which epigenetics are dynamic,^21^ an effect potentially observed by Munesue et al. whose only population subgroup that experienced positive effects from oxytocin treatment were the youngest participants.^102^ A connection between exogenous oxytocin administration and methylation status has been recently demonstrated in, Moerkerke et al. This double-blind, randomized, placebo-controlled study demonstrated that chronic intranasal administration of oxytocin decreases *OXTR*m.^103^ This finding demonstrates the ability of exposure to oxytocin to regulate methylation status of the receptor gene and highlights the importance of understanding how exogenous oxytocin applications interact with the epigenetic profile.

### A mechanistic understanding of oxytocin’s benefits can inform future *OXTR*m research and therapies

It is well known that oxytocin impacts the social brain; however, the mechanism by which oxytocin impacts neuronal scaffolding on the molecular level is not well understood. A fine-grained understanding of oxytocin’s impacts on the developing brain can help establish precise therapeutics. Early inflammation is one mechanism through which neural development can be disrupted, and is a process that may be mitigated by oxytocin.^104–106^ Mairesse et al., for example, found that microglial activation of Oxtr regulates inflammation in the neonatal rat brain.^105^ Similarly, Kingsbury et al. found that application of an Oxtr agonist ameliorated the impact of early life inflammation by preserving myelination and functional connectivity.^106^ These results point to a neuroprotective mechanism whereby Oxtr may modulate early levels of inflammation and therefore impact later outcomes. For instance, Ceanga et al. found improved viability of neural tissue incubated with oxytocin under hypoxic conditions, which was not observed when Oxtr is blocked with an antagonist.^104^ These findings have potential implications in human infants experiencing inflammatory events such as those induced by hypoxic stress.

Another avenue of research includes OXTR’s role in the “GABA switch”. Early in development, the most important inhibitory neurotransmitter, Gamma-aminobutyric acid (GABA), is instead excitatory.^104,106–108^ This excitation is critical for the formation of early life neuronal circuitry and growth.^107,108^ However, if GABA’s actions are not “switched” to inhibition within early development, individuals are left with cognitive deficits mimicking those observed in ASD.^109^ GABA’s excitatory effect in the developing brain has been attributed to an increase in the neuronal sodium-potassium-chloride cotransporter NKCC1, which is responsible for concentration of intracellular chloride. As the fetus develops, the chloride transporter KCC2 is upregulated in the cell membrane, allowing chloride to exit the cell; this net decrease in intracellular chloride allows GABA to become an inhibitory neurotransmitter.^107^ Oxtr is key in both downregulating the NKCC1 receptor and upregulating the expression of KCC2.^107^ This “GABA switch”, which persists through maturity, requires precise timing, which is directly regulated by Oxtr.^107^ *Oxtr* knockout mice lacked a significant increase in transcription of the KCC2 receptor while mice with *Oxtr* experienced a five-fold increase in transcription of KCC2 at the time of the GABA switch.^107^

OXTR’s dominant role in the GABA switch has provided key insights into the neuronal etiology of ASD, as well as a potential target for treatment. A delayed or absent GABA switch is seen in many animal models of ASD, including the valproate and Fragile X rodent models.^110,111^ Research into the electrophysiology of these models have shown hyperexcitability in response to GABA administration, which is also seen in humans diagnosed with ASD.^112^ Tyzio et al. found that the GABA switch is completely absent in both of these murine ASD models.^110^ When the NKCC1 receptor is blocked in pregnant mothers, simulating the GABA switch by downregulating intracellular chloride concentration, the behavioral control phenotype was restored in their pups. Conversely, when researchers blocked activity of Oxtr in pregnant dams, their pups displayed similar hyperexcitation and behavioral interactions as the murine ASD models.^110^ Bertoni et al. similarly found both a delay in the GABA switch and disrupted social memory in the Magel2^tm1.1Mus^ mouse model of ASD; however, these aberrancies were reversed when oxytocin was injected in neonatal mice.^113^

Critically, Bertoni et al. also found that early application of oxytocin (postnatal day 0) altered Oxtr expression in adulthood.^113^ This finding mirrors the data described by others which showed plasticity of *OXTR*m in infancy^21^ but relative stability in adulthood.^21,114^ Together, these findings shape the hypothesis that DNA methylation of the oxytocin receptor could be a potent therapeutic target for influencing the oxytocinergic system throughout the lifespan.

These studies have elegantly shown that Oxtr is necessary to facilitate the GABA switch and that decreasing expression of this protein could impact excitatory/inhibitory (E/I) ratios throughout the lifespan.^115^ Oxytocin therapeutics, therefore, have the potential to shape early neuronal scaffolding.

An understanding of the impact of *OXTR* and oxytocin on the establishment of neuronal circuitry early in life allows researchers and clinicians to investigate how these relationships could be manipulated to support positive social outcomes, as outlined in Fig. 4. Research in mice has shown that maternal separation was sufficient to delay the GABA switch and produce adverse behavioral consequences.^116^ We believe this finding to be particularly relevant to the care of children born preterm who experience high rates of early maternal separation and who are at increased risk of ASD.^117,118^ The use of GABA therapies in the Neonatal Intensive Care Unit has been gaining traction in recent years. Gabapentin, a molecular analog to GABA, has been traditionally used to treat seizures, but its efficacy in soothing neonatal agitation and pain has been shown in several recent publications.^119,120^ At this time, there is no published research demonstrating a therapeutic intervention intentionally targeting *OXTR*m in humans or using GABA-therapies to impact neural circuitry development; however, we believe this vein of investigation has immense therapeutic potential.

## Limitations and conflicting results in *OXTR*m literature

It must be acknowledged that many *OXTR*m studies demonstrate conflicting results. We believe that these discrepancies can often be explained by methodological differences across studies that can be addressed in future research. For instance, some studies failed to correlate *OXTR*m with the early life environment,^67,121,122^ while others found weak, null, or opposing results when examining *OXTR*m as a link between early life experience and later psychological outcomes.^123–125^ Robakis et al. assayed over 1000 regions where methylation density could be associated with early life adverse experiences; however, found no associations between *OXTR*m and psychological outcomes following early life adverse experiences.^126^ Likewise, Parianen et al. found that *OXTR*m was associated with decreased neural activity in response to facial expressions, but this finding did not survive statistical correction.^127^

Despite these conflicting findings, we believe that there is potential for resolution with continued research. For example, the Illumina MethylationEPIC 850k array, a common tool for assaying methylation that powerfully probes almost one million CpG sites across the entire epigenome, contains 22 sites from *OXTR*, however does not contain the sites that have been shown to be functional in the gene’s regulation (Fig. 1).^16,18,128^

Additionally, some opposing results could be explained by hydroxymethylation, an epigenetic mechanism within the methylation cycle that occurs when a methylated cytosine is oxidized. While this modification was previously thought of as transient, it has been shown to impact gene regulation by *promoting* gene transcription.^129,130^ Hydroxymethylation clouds interpretation of methylation results because it is indistinguishable from “true methylation” using conventional techniques such as bisulfite conversion^131^ and therefore requires its own assay.^129^ As a result, methylation levels determined by bisulfite conversion are a combination of hydroxymethylation and “true methylation”.

Finally, it is important to consider age and tissue differences as a potential explanation for discrepancies across studies. Although significant correlations in *OXTR*m levels have previously been demonstrated across tissue types,^21–23^ there may be divergent developmental and/or temporodynamic differences in these tissues that are not captured by these correlational assays in limited age groups. For example, a study of salivary cellular content across age groups found that children’s saliva contained a significantly higher proportion of buccal epithelial cells than adults’ saliva.^132^ This difference may be significant for using saliva as a proxy for the brain—the causal tissue for behavior—in epigenetic research because both buccal epithelial cells and neurons are derived from the ectodermal layer during development.^133^ Furthermore, blood *OXTR* methylation levels may reflect systemic, trait levels that can account for individual differences in established brain network patterns whereas other tissue types may provide a more plastic and dynamic understanding of *OXTR*m variability over time. Future longitudinal work that tracks associations between the early life environment, *OXTR*m, and social-behavioral outcomes from infancy to adulthood in the same tissue is necessary to distinguish whether developmental or methodological factors account for discrepancies in the literature.

## Conclusion and future directions

The field of oxytocin therapeutics research is primed to inform novel pharmacological and behavioral interventions aimed at populations who are at increased risk for neurodevelopmental disorders. This review demonstrated that oxytocin receptor epigenetics are malleable in infancy, related to subsequent neurodevelopmental outcomes, and may inform precision medicine and “precision parenting” approaches and serve as powerful therapeutic targets. A deeper knowledge of *OXTR*m will allow researchers to target the most relevant sites of *OXTR* and their methylation through manipulations to the early care environment and/or the functional, cellular mechanisms that tune *OXTR*m to harness its plasticity and improve the neurodevelopmental outcomes of high-risk infants and children.
